# Ichthyol in Erysipelas

**Published:** 1905-03

**Authors:** 


### ICTHYOL IN ERYSIPELAS.

This is one of the most interesting drugs, and yet it is not in such common use as its virtues warrant. It is prepared from an oil which is obtained by the destructive distillation of fossilized fish found in the Tyrolese mountains. No one has yet given any rationale of its action, yet Hare declares it "the best external treatment of erysipelas that we have." In employing it in erysipelas, the skin is well washed, and then anointed with the following prescription :

| | |
|---|---|
| Ichthyol | 1½ oz. |
| Oil citronelle | 20 drops. |
| Lard or vaseline | 1 oz. |

and is then covered with lint upon which the same ointment has been spread. It is also valuable as an inunction in acute rheumatism, and the same prescription is serviceable. In chronic skin diseases characterized by atony and induration of the deeper layers of the skin, it is of marked benefit. Properly rubbed in, in case of acute sprains, its effect is almost magical. In fissured nipples, accompanied by induration, an ointment containing one part of ichthyol to four of lanolin will give prompt relief; it must be carefully wiped off the nipple before each nursing, as the "tarry" odor will frequently cause the child to refuse the breast. Agnue esteemed it highly in cases of lymphatic enlargement.—*Indiana Medical Journal.*

